# Pharmaceutical Pollution and Disposal of Expired, Unused, and Unwanted Medicines in the Brazilian Context

**DOI:** 10.3390/jox11020005

**Published:** 2021-05-18

**Authors:** Letícia de Araújo Almeida Freitas, Gandhi Radis-Baptista

**Affiliations:** 1 Post-Graduate Program in Pharmaceutical Sciences, School of Pharmacy, Dentistry and Nursing, Federal University of Ceará, Fortaleza, CE 60416-030, Brazil; leticiaraujo@edu.unifor.br; 2 Laboratory of Biochemistry and Biotechnology, Institute for Marine Sciences, Federal University of Ceará, Fortaleza, CE 60165-081, Brazil

**Keywords:** pharmaceutical pollution, drug pollution, active pharmaceutical ingredients, environmental pollution, hazardous waste, drug disposal, xenobiotics

## Abstract

The occurrence of pharmaceuticals in the environment is an everyday recognized concern worldwide, and drugs as environmental contaminants have been detected in water and soil systems, posing risks to humans and wildlife. The presence of drugs in wastewater, groundwater, and even drinking water occurs in several countries, including Brazil, where the pharmaceutical market is expanding over the years. The adverse, harmful effects of pharmaceuticals in the environment range from the spreading of antimicrobial resistance and species survival to the interference with reproduction and increased cancer incidence in humans. Therefore, it is demanding to count on proper legislation to prevent these pollutants from entering the distinct environment compartments. In some developed countries, laws, directives, programs, and initiatives regarding drug disposal reach a mature status. In Brazil, federal laws dealing with drug residues’ management are recent, with flaws that might facilitate non-compliance with drug pollution issues. Besides, pharmacies and drugstores are not obligated to collect unneeded household medicines, while particular State laws aim to ordinate the disposal of drug residues regionally. In this review, we consider the current knowledge about pharmaceutical (drug) pollution, the recommendation and regulations on the disposal of useless medicines in some countries, and in the context of the expanding pharmaceutical market in Brazil. The awareness of emerging contaminants in the environment, besides the joint effort of authorities, consumers, and the general public nationwide, will be required to avoid pharmaceutical/drug pollution and achieve an eco-friendly environment and a sustainable society.

## 1. The Pharmaceutical and Healthcare Market and Industry in Brazil

By a recent report of the Association of the Pharmaceutical Research Industry (INTERFARMA, Brazil), a non-profit association that represents almost 50% of all Brazilian pharmaceutical retail, which comprises drugstores and pharmacies, the revenues in the Brazilian pharmaceutical market increased from US$21.89 to 80.6 billion, from 2005 to 2018, This market volume reached a cumulative revenue of US$586.6 billion, as estimated by the Brazilian Medication Market Regulating Chamber. The Brazilian pharmaceutical trade is expected to reach a value of over US$40 billion by 2023, exclusively regarding prescription, over-the-counter, generic, and similar medicines. In Latin America, Brazil is one of the main markets, besides Mexico, Colombia, and Argentina, with more than two hundred regularized pharmaceutical laboratories. Such data and estimates point that Brazil can reach a position among the top five major global pharmaceutical markets currently led by the U.S., China, Japan, and Germany [1]. Globally, the Brazilian pharmaceutical trade and industry is only a fraction of an anticipated US$1.5 trillion global market by 2028 [2], but still a significant growing market with a large horizon to expand. In this context, higher sales and consumption imply higher quantities of chemicals entering the environment. Only in São Paulo city, the region most economically active and with the highest gross domestic product in Brazil, several metric tons of non-steroidal anti-inflammatory drugs, anti-diabetic and antihypertensive drugs, and steroid hormones have been consumed in the last years [3]. In Brazil, among the most commercialized medicines, muscle relaxants and medications to treat deep venous thrombosis leads to internal sales (Table 1), in a commercial volume that exceeds the third-place medicine ranked among the best-seller drugs in the world.

## 2. Pharmaceutical (Drug) Pollution

In such an expanding pharmaceutical and healthcare market and industry, in Brazil, drug pollution emerges as an unprecedented local and worldwide public health concern, even if it is not perceptible as an environmental and health threat by the general public [4]. Besides the steadily bulky discharges of untreated or inappropriately treated industrial wastewater, pharmaceutical pollution includes the active ingredients and the final medicinal products (micropollutants) discarded by residences and healthcare systems [5,6]. Even illicit drugs and their metabolites related to drug abuse, like cocaine, methamphetamine, opioids, ketamine, benzoylecgonine, among others, have been detected in wastewaters of several urban localities, contributing to increasing the problem with water quality [7,8].

One of the most significant environmental and public health concerns is related to the active pharmaceutical ingredients (APIs) and medicines discharged in the water bodies. This preoccupation is because their hazardous concentrations do not necessarily follow a typical dose-response curve of toxicity, by which their cumulative quantities of contaminant could cause a deleterious effect [9]. Notably, this is the case of the endocrine-disrupting chemicals (EDCs) that are harmful to wildlife and humans at low, minute concentrations. Other factors that make environmentalists’ and scientists’ concerns alarming are the potential persistence of APIs in the environment and their metabolism, which occurs through biotransformation and can increase and perpetuate these bioactive substances’ harmful effects on the environment [10]. Thus far, the emission of APIs/PPCPs as pollutants in the environment is a critical issue that needs to be evaluated and widely discussed because of its inherent local and global relevance. Significantly, this issue relies on the fact that attention is usually focused on environmental pollution caused by large quantities and volumes of hazardous residues from chemical and processing industries and final products, like plastics, rather than the APIs. The healthcare industry’s expansion correlates with a concomitant increase in the volume of pharmaceutical waste due to the increase in the number of consumers and patients, consumption of over-the-counter and prescription medicines, and excessive production of medications [11]. Moreover, the increase in the production, diversification, and consumption of PPCPs, which includes facial creams and cleansers, sun blockers, repellents, fragrances, among others, contribute to the overall concern and overload of APIs/PPCPs in the environment that could result in drug contamination and pollution [12].

### 2.1. Contamination of Soil and Water by Pharmaceuticals

The APIs and API-containing products enter into the water systems through diverse routes, for example, human and animal excretion and disposal through flushing the toilet. Because these bioactive chemicals are not entirely eliminated by conventional sewage treatment, they accumulate in the aquatic environment. Loper and colleagues [13] reported diverse pharmaceutical compounds, including antibiotics, in water samples from streams and wells that received municipal wastewater and runoff from agricultural areas in which animal-feeding operations are active. Muscle relaxants and antibiotics predominate in water samples from streams and wells, but also detected in high concentrations were anti-histamine (diphenhydramine), anti-inflammatory (e.g., ibuprofen), stimulants (caffeine and metabolites), and alkaloids (cotinine). In another study by the U.S. Geological Survey (USGS), high concentrations of pharmaceutical-related chemicals, like therapeutic opioids and muscle relaxants, were detected in wastewater treatment plants (WWTPs) that received discharges from pharmaceutical formulation facilities in comparison with effluents from other localities [14]. At the U.S. Environmental Protection Agency (U.S. EPA), a workgroup on emerging contaminants elaborated a document about the challenges and recommendations regarding chemicals of emerging concern (CECs) in aquatic ecosystems. Several classes of chemical contaminants that included PPCPs and EDCs, among others, are reported in this document [15].

Another examination in this regard is from a deep study that reported the assessment scales and trends of pharmaceuticals’ presence in the environment. Therein, the potential impact of pharmaceuticals in the environment, the legal issues, and current practices that could influence or restrain environmental contamination by medicines was analyzed [16]. In European Community (E.C.), the European Self-Medication Industry, the European Federation of Pharmaceutical Industries and Associations, and the European Generic and Biosimilar Medicines Association have committed to assuring a minimal pharmaceutical release into the environment [17]. In agreement with these issues, the European Commission has, among its directives, established strategies to control effluents from the pharmaceutical manufacturing facilities and industries [18]. Nowadays, an increasing number of studies regarding the investigation on the occurrence of APIs in the environments, particularly in water bodies, with the studies mentioned above, give a glimpse about the recognized emerging presence of APIs/PPCPs in wastes, soil, sewages, coastal seawater, pounds, rivers, groundwater, and even aquifers and drinking water, in urban areas and remote localities in the planet [19,20,21].

Zuccato and colleagues [22] analyzed several aspects of drug pollution, such as the occurrence, monitoring, treatment, control of the emissions, and pharmaceuticals’ effects on Italy’s environment. In marine coastal waters and in waste and river waters that flow into the Mediterranean Sea, the levels of hormones (17α-ethinylestradiol and metoprolol), antibiotics, analgesics, and anti-inflammatories among more than 40 substances assessed from different classes of pharmaceuticals, were of the highest concern based on the correlation between occurrence and ecotoxicology, as determined by the Hazard Quotient, HQ [23]. Li et al. [24] also addressed the occurrence, sources, and fate of some examples of pharmaceuticals detected in aquatic environments and soil worldwide. In the Henan Province of China, the occurrence of several target pharmaceuticals and other chemical micropollutants in aquatic environments indicated a high risk to the components of the aquatic ecosystem as indicated by risk quotient (RQ) analysis. Fortunately, a relatively negligible risk to humans was estimated by HQ calculation [25]. In India, Balakrishna and collaborators [26] compiled several studies about pharmaceuticals in the aquatic environments, like influents of wastewater treatment plants, hospital effluents, surface-water of rivers, and groundwater. Some kinds of psychoactive drugs, antihypertensive, antimicrobials, and analgesics were detected at concentrations higher than found in North America, Europe, and Asia; despite this, these chemical substances were qualitatively similar to those found in China. In a report by the Irish Environmental Protection Agency, published as part of the Research Program 2014–2020 on chemicals of emerging concerns, several issues regarding pharmaceuticals in aquatic environments were considered to unify the knowledge about the occurrence, distribution, fate, and toxicological effects in aquatic organisms, as well as the potential risk to human health and the methods of sampling and analysis of pharmaceuticals in water pools and wildlife [27]. Even in remote, pristine localities, as is the case of the Artic, hundreds of substances related to the PPCPs, released primarily by domestic and municipal wastes and sewage, were detected in diverse environments from the coastal seawater, and several organisms that compose the wildlife [28].

In Brazil, pharmaceuticals like hormones and anti-inflammatory agents have already been detected decades ago in raw sewage, treated wastewater, and river water in Rio de Janeiro (Brazilian southern east) [29]. In another study, the estrogenic potential of water sampled from different depths in the nearshore marine environment in Rio de Janeiro, as determined by the yeast estrogen screening assay, indicated that EDCs were adsorbed in the sediments [30]. Moreover, the surface water of a river tributary of a lagoon system in the oceanic region of Rio de Janeiro, which suffers from the discharge of untreated urban sewage, presented extreme ecological risk indexes associated with high concentrations of the steroid 17α-ethynylestradiol and the antibiotic sulfamethoxazole [31]. In treated water and surface water of suburban rivers in Brazilian inland, steroid hormones (estriol, estrone, 17β-estradiol, 17α-ethinylestradiol) were present in concentrations that could compromise the aquatic biota [32]. A comprehensive study about pharmaceuticals as contaminants in Brazilian water bodies pointed that 17α-ethinylestradiol, 17β-estradiol, and caffeine predominate in surface waters. At the same time, other APIs and PCPPs occurred at low concentrations, but that caused deleterious effects in aquatic biota as observed by ecotoxicological assays [33].

Psychiatric medicines, particularly benzodiazepines, were detected in municipal public water supply in Rio de Janeiro at relatively high levels to pose ecological and health risks [34]. In sewage effluents of a University Hospital in South Brazil, diverse psychoactive (anti-anxiety and anti-epileptic) drugs, particularly carbamazepine and diazepam, were found that posed environmental risk of toxicity once untreated wastewater is directly discharged into the water stream [35]. Roveri and collaborators [36] investigated the occurrence of several APIs that included psychoactive drugs and illicit drugs and their metabolites in the seawater in the vicinity of coastal submarine sewage outfalls in different seasons and water column depths. Among the drugs found, the high ecological risk was associated with caffeine, diclofenac, and acetaminophen. Neves and Mol [28] evaluated the theoretical environmental risk for ten of the most used medicines in the Public Health Service in Minas Gerais State’s capital in Brazil based on risk quotient estimates. Accordingly, they concluded that these selected pharmaceuticals, which included the psychoactive drugs clonazepam and losartan, were potentially harmful to aquatic life based on the environmental risk assessment criteria. Santos and colleagues [37] investigated the occurrence and the environmental risks of twenty-eight APIs during one year in four Brazilian water sources. They noticed a correlation between seasonality and socioeconomic aspects. The drug pollutants most commonly detected in the highest concentration were betamethasone, prednisone, and fluconazole, whereas those with the highest toxicological risks were loratadine, atorvastatin, norfloxacin, caffeine, and ranitidine.

Studies on the deleterious effects on wildlife caused by the presence of APIs and PCPPs in water matrices in Brazil include, for example, the alteration of reproductive parameters, cytogenotoxicity effects, and lysosomal membrane stability in brown mussel *Perna perna*, caused by the antihypertensive drug losartan in seawater [38]; alteration of gene expression in experimental zebrafish *Danio rerio* embryos with the increasing of EDC concentrations in Brazilian rivers [39]; bioaccumulation of cocaine and benzoylecgonine in brown mussel tissues due to the occurrence of these drug pollutants in marine water and sediments [40]. The presence of pharmaceuticals and hormones in untreated wastewater and their metabolites in treated and post-treat sewage effluents interfered with several biochemical parameters (e.g., oxidative damage and enzymatic activities) in the experimental freshwater tetra fish *Astyanax bimaculatus* [41]. Besides, various PPCPs in water reservoirs in Brazil were documented, despite a variable level of removal efficiency achieved through ecological filtration for a specific contaminant class [42]. These study examples give a glimpse into the emerging occurrence of pharmaceuticals in distinct localities and their latent presence in the environment to pose a risk to human health and wildlife.

In Table 2, some APIs and PCPPs found in several kinds of water bodies and sediments in Brazil are summarized.

### 2.2. Harmful Effects of Pharmaceutical Pollutants in Water Bodies

Pharmaceuticals are biologically active substances of which the occurrence in distinct environmental compartments, particularly water systems, and the potential health risks they pose to living organisms, including humans, cannot be underestimated. Usually, effluent treatment in conventional WWTPs is not sufficiently efficient to remove all physicochemically diverse APIs discharged in wastewaters. The efficiency of pharmaceutical and chemical removal varies according to the technologies of wastewater treatment, and these technologies do not work well to remove the entirety of bioactive chemicals from sewage effluents [7,10]. For instance, Komolafe et al. [53] investigated the occurrence and effectiveness elimination of several chemicals from different classes (e.g., triclosan, polycyclic aromatic hydrocarbons, estrogens, and polybrominated diphenyl ethers), ranging from 0.1 to 49 μg L^−1^, in three technologically diverse full-scale WWTPs. These technologies comprised conventional activated sludge (CAS), waste stabilization ponds (WSPs), and up-flow anaerobic sludge blanket reactors (UASBs) and represent the leading technologies used for wastewater treatment in Brazil. Accordingly, the wastewater treatment system that best performed for such bioactive chemicals was the WSP with an 89–99% removal efficiency, comparable to CAS. However, despite the considerable high capacity of removal achieved by all three waste treatment systems, the residual effluent concentrations of bioactive compounds (triclosan and estrogens) were above their environmental quality standards, posing the aquatic biota at potential risk [53]. These facts are of crucial importance concerning pharmaceutical pollution nationwide. Brazil, which reached a population of over 213 million in 2020 and has one of the most expressive extensions of coastal seawater (~8000 km) and resources of surface freshwater globally (~12% of the total), counts with less than 3000 WWTPs. In terms of the number of WWTPs in operation, the configuration most commonly adopted is an anaerobic pond followed by a facultative pond. Another design of WWTPs operating in Brazil comprises the UASBs followed by CAS [55]. These WWTPs account for only 40% of sewage treatment, based on the volume of sewage treated to the volume of water consumed, or approximately 70% if it is considered the volume of sewage treated regarding the volume of sewage collected [55,56].

Hence, collective efforts and strategies are required to avoid the emission of APIs/PCPPs and their increasing dissemination in environments in their active forms, aiming to avoid short- and long-term adverse effects on the biota and human health [10]. The range of reported effects resulting from pharmaceutical pollution includes the harmful risk to wild organisms, the emergence and dissemination of antimicrobial-resistant bacteria, the interference on the reproduction, development, and survival of species, and the increase of cancer incidence in humans, to mention the most known examples.

For instance, EDCs, which comprise a broadly diverse group of chemicals, in addition to some PPCPs, mimic hormones in their structures and activities and interfere with human and biota physiology. EDCs comprise natural and synthetic estrogens, alkylphenols used in detergent products, bisphenol A used for the lining of food and beverage cans, phthalates (plasticizer agents), organochlorine pesticides, perfluorinated compounds used as components of fire-fighting foams, polychlorinated biphenyls used as insulators in electric equipment, among others [57,58]. These substances, as environmental contaminants, interfere with hormone signaling and the endocrine system by mimicking natural hormones, causing immunologic, metabolic, neurological, and reproductive adverse effects in wildlife and human populations once ingested. These adverse effects range from amphibians and fish femininization to cancer and obesity in humans [57,58]. Steroidal estrogen contaminants, with estimated discharges of over 100 tons per year, are detected in wastewater effluents and even groundwater and are involved in the interference of reproduction, development, and physiology of organisms as diverse as fish and plants. In humans, estrogens and their metabolites, ingested through drinking water and food, are associated with an increased risk of certain types of cancer, like breast cancer and the reproductive system’s disorders [59].

The emergence of antibiotic resistance of microorganisms is another adverse effect caused by APIs in the environment. Nowadays, the expressive consumption of antibiotics worldwide is proportionally associated with thousands of metric tons of antimicrobials and related substances released in the environment, mainly through wastewaters, resulting in antimicrobial resistance. Due to antibiotic and drug pollution, antimicrobial resistance is an increasingly recognized problem that contributes to disseminating clinically relevant multidrug-resistant microorganisms that are difficult to treat in clinical settings [60]. Identifying the predominant contaminants (drivers) and pathways leading to the emergence of antimicrobial resistance in the environment limits its occurrence and gives policymakers and environmental regulators support to elaborate plans and take actions [61,62]. Large-scale human and veterinary antibiotic use, with consequent release in the municipal and industrial wastewater, and the spreading of animal manure and sewage sludge on land are the major contributors to antimicrobial resistance due to pharmaceutical pollution. In surface waters, antibiotics can persist for days to months [63]. The emergence and dissemination of antimicrobial resistance in the environment threaten globally and, severely, human and animal health and harm nations’ economic sustainability due to the present antibiotic crisis.

Moreover, questions have arisen whether drivers (antimicrobials, APIs, biocides, PCPPs, chemical pollutants, and stressors) that were and have been released in the environment could effectively be controlled now and in the future [64]. Indeed, the awareness of the occurrence and abundance of antimicrobials and drivers in distinct compartments of the environment, as well as the pathways that cause adverse ecotoxicological effects in the aquatic environment as a consequence of these contaminants and drug pollutants, allows the authorities and communities to counteract the growth and dissemination of antimicrobial resistance traits among microorganisms [63]. Furthermore, the increasing occurrence of antibiotics of human and veterinary use in the environment and food chain has also been implicated in the present-day epidemic of obesity. Even though this is a less known adverse effect caused by antibiotics in the environment, pharmaceutical pollution due to antibiotics can trigger an obesogenic effect in humans [65].

The extensive use of diclofenac, a non-steroidal anti-inflammatory drug (NSAID), in veterinary medicine has caused a decline in the vulture population in the Indian sub-continent and placed three keystone Asian species of vulture at risk of extinction. As a result of scavenging dead livestock previously treated with such an NSAID, diclofenac’s intake causes a collapse in several internal organs of the vulture (visceral gout) and subsequent vulture’s death [66]. Thus, pharmaceuticals in the environment affect not only species in aquatic systems but also non-target biota in terrestrial environment compartments, requiring changes in veterinary pharmaceuticals to pose critical species at risk and cause ecological disturbance [66,67]. The effect of pharmaceutical contaminants on wildlife, additionally, includes the direct and indirect ecotoxicological behavioral alterations in fish (and related prey) exposed to increasing levels of psychiatric drugs (benzodiazepines) in freshwater systems [68]. These are few but dramatic examples of adverse effects of pharmaceutical pollutants on non-target organisms in different environmental compartments. For further insights into the occurrence of APIs/PCPPs in the environment and their effects, interesting literature can be further consulted [6,10,24,69].

## 3. Disposal of Expired, Unused, or Unwanted Medicines

Invariably, medicines purchased and maintained by consumers are unintentionally accumulated in their homes and become useless for different reasons, like expired date, the excessive amount due to changes in the treatment or dosage regime, prescription in excess or excessive dosage, preservation for presumable usage in the future, over-the-counter medicine retailers and leftover from finished treatment, to mention a few [70]. Indeed, in most places of the world, these leftovers are commonly and inappropriately discarded by citizens in the ordinary garbage and sewers, as lack of own personal attention, lack of understanding, knowledge, and education, or even by absence of proper governmental and healthcare authorities’ directions and regulations [11]. Controlling the discharge of pharmaceutical pollutants in the environments, particularly aquatic systems, requires different strategies and technologies, like, for example, the commitment of manufacturers to avoid the emission of harmful APIs, application of green-chemistry principles for production process and remediation, and medicine-disposal and educational campaigns, like reverse logistic, and regulation for consumers and traders [11,71,72]. In an attempt to draw guidelines for the proper disposal of drugs that become useless, many developed countries have already established some strategies, programs, and regulations to minimize the pharmaceutical pollution risk posed to the environments caused by the improper disposal of expired, unused, or unwanted medicines [11,72]. Despite international attention on pharmaceutical pollution as a growing environmental threat, locally, the subject’s legislation is still unfinished in Brazil. Only draft directives are under consideration and waiting for a final definition by vote since the last decade in the lower deputy house [73]. However, few isolated actions by private institutions and governmental authorities in some Brazilian states and cities joined efforts to collect and handle this category of micropollutants.

### 3.1. Collecting Schemes and Regulation on Drug Disposal Around the World

The U. S. Food and Drug Administration (FDA) provides in its webpage information and instructive directions to safely dispose of unused, unwanted, or expired medicines through the take-back program or flushing down the toilet if the useless medicine is on an elaborated list (the FDA “flush list”). According to FDA criteria, Table 3 lists the APIs in medicines allowed to be discarded by flushing. FDA recommends, firstly, to find the take-back localities to collect both prescription and or over-the-counter medicines for disposal. There are two take-back options: the permanent collection locations and sites, and the periodic events (the National Prescription Drug Take-Back Days), both with the U. S. Drug Enforcement Administration (DEA) collaboration. If the take-back option is unavailable, customers can discard the useless medicines by flushing the toilet if these are on the FDA “flush list.” If not, then it is recommended to discard useless pharmaceutical products in the household trash, taking care of the following simple steps for safe disposal, like, for example, mixing them with inert, unpalatable material (e.g., coffee ground), place the mixture in a sealed container (e.g., Ziplock plastic bag) and thrown away in the trash [74].

Take-back programs and collecting schemes are acceptable strategies to avoid medicines ending up in the environment, but even in European Community, these are not a consensual, general practice in all E. C. Member States. For instance, in a snapshot of European Collection Schemes by the Health Care Without Harm (HCWH) Europe, on unused pharmaceuticals, involving six Member States, it was pointed that, despite a unified directive (2004/27/EC) to implement appropriate collection schemes for pharmaceutical residues, standard guidelines are lacking to place in practice collecting schemes or take-back programs. So, contrastive differences to safely dispose of unneeded medicines among the E.C. Member States are evident, ranging from the absence of means to deal with medicine residues to rational “green pharmacy,” in which practices on production, prescription, and waste disposal are accomplished [75]. Besides, in Europe, a joint initiative of European healthcare, industry, and student organizations maintain an interactive platform regarding a campaign to draw attention to the importance and the mode of the proper disposal of expired, unused, or unwanted drugs. Recommendations are State Members-based, and further information is usually available by consulting authoritative local organizations (http://medsdisposal.eu/ (accessed on 17 May 2020)). Although the take-back schemes contribute to the population’s engagement regarding the controlled disposal of medicines and flushing down useless medicines in the toilet or discard them in the trash appear safe, concerns arise about the health risk of harmful effects of APIs released in the environment. The emissions of APIs and related products in the environment, according to the final disposal method (take-back program, household waste, or clean flush), are not negligible and environmental contamination (water, soil, and even air) may occur at variable levels [76]. Despite this observation, all of the fifteen APIs currently on the FDA “flush list” presented negligible ecological and human health risks associated with water and fish ingestion [77].

In Brazil, although health scientists are aware of the importance of take-back options and collection schemes, endorsement and support by public authorities are lacking, and any alternatives exist [71]. In this regard, Oliveira and collaborators [78] reviewed the international and national regulations on managing drug residues to take current directions. They confirmed that examined countries in Europe, the Americas, and Australia all count with strict governmental regulations on drug management (e.g., registration grant connected with environmental risk assessment, ERA), as well as programs for safe disposal of unneeded medicines, and programs for aware the ecological and human health risks related to the inadequate disposal of medicines in the environment, as aforementioned. Importantly, they pointed that in Germany, the U.S., and Sweden, ERAs are mandatory for drug registration. In Sweden, data concerning the ERA of pharmaceutical residues need periodical updates. The Federal Environment Agency of Germany requires that manufacturers report the ERA data to register medicines for human and veterinary use.

Moreover, disclosed information in their respective packaging about the environmental risks of medicinal products is demanding. However, in German, the collection of unused drugs by local pharmacies is optional, and the disposal of unwanted drugs in domestic waste is allowed. In Sweden, the ERA data of APIs are a tool for medication management. The environmental hazard of drugs relates to the PBT index of each drug or its active principle, corresponding to the integration of its persistence (P), bioaccumulation (B), and toxicity (T). Thus, a list of essential medicines (Kloka Listan) is periodically updated, including the drug efficacy and cost-benefices and the environmental impact they might cause. Olivera group’s review [78] then supported that these international regulations and programs for drug registration, associated with ERA data and safe management of drug residues, as well as awareness of ecotoxicological and human health risks of medicines and APIs, could serve as references for ethical and technical discussion, educational campaigns, elaboration of legal directives and adaptations or amendments in regulations concerning to drug disposal in Brazil.

### 3.2. The Governmental Regulation about the Disposal of Drugs in Brazil

In Brazil, specific regulations on waste management encompass, in general, specific sectors of the pharmaceutical production network (directive RDC No. 306/2004 of the National Agency of Sanitary Surveillance, ANVISA and No. 358/2005 of the National Council of the Environment, CONAMA), which refer to the management, treatment, and final disposal of waste produced by health services. The ANVISA RDC No. 17/2010 and No. 222/2018 provide directives for good manufacturing medicines practices and good healthcare waste management practices. It is important to note that these directives do not regulate a medicine residue’s final disposal in its liquid forms but recommend disposing of them in an appropriate landfill after converting them into solid pharmaceutical waste. Despite this, these directives do not discriminate all pharmacological classes of medicines for human use and veterinary use drugs. In addition to this regulation (RDC No. 222/2018), another resolution (RDC No. 44/2009) addresses the disposal of medicines in pharmacies and drugstores. However, these regulations do not refer to expired or unused drugs’ domestic disposal. At best, it would comprise a shared responsibility for the disposal of medicines among market players involved in the production, distribution, sale, and consumption. Thus, it is presumed shared participation for the proper disposal of generated drug wastes, tentatively avoiding polluting the environment. There is a Brazilian directive that addresses the exclusive participation of consumers in the drug disposal process; this establishes the rule for the destination of unused controlled drugs (Ordinance 6/1999). In such a case, local health authorities should receive and handle controlled drugs, expired or out of use, for proper disposal.

According to the National Solid Waste Policy, the demands and responsibilities should observe the reverse logistics of manufactured products, except for useless medicines. Such gaps and imperfections observed in these directives regarding expired, useless, and unwanted medicines show the necessity to improve the shared responsibility of reverse logistics and appropriate disposal of useless product wastes exclude pharmaceuticals.

Recently, a published normative (Decree nº 10.388) regulates the reverse logistics of medicines, expired or unused, from Brazilian homes. However, this regulation does not encompass the medicines for veterinary uses and the personal care products, cosmetics, and dermato-cosmetics. Another flaw in recent regulations is restricting drug disposal procedures only to cities with over a hundred thousand inhabitants. The pharmacies and drugstores in smaller municipalities are not obligated to comply with reverse logistics to collect expired or out-of-use home medicines. Nor do they participate as voluntary point collection of unwanted medicine. Apart from federal laws and regulations, there are specific state laws in most Brazil regions regarding (Supplementary Table S1) to cope with unwanted medicines disposal. These comprise variable and locally restricted regulations to a total absence of directives.

## 4. Conclusions

Pharmaceutical pollution is an emerging public health concern worldwide associated with the increased production and consumption of pharmaceutical and healthcare products. The fact that not only the discharge of high volumes of bulk chemicals and raw materials but also the inappropriate disposal of active pharmaceutical ingredients from medicinal and personal care products can be detrimental to the environments even at low concentrations demand joint public efforts to deal appropriately with medicine residues, through regulations, technical directions, and educational campaigns nationwide.

In the absence of appropriate directions and programs for the destination of drug residues, public health and wildlife in diverse biomes will be at risk. Thousands of tons of different pharmaceutical classes of substances enter the environments by distinct routes and, some countries permit that citizens throw in the sewer expired or unused medicines. Consequently, the water systems, including groundwaters, are the final destinations. Conventional WWTPs cannot remove all APIs efficiently, and some procedures can metabolize these APIs to more active substances. Therefore, these pharmaceutical pollutants can persist and contaminate the water and soil systems for long life cycles. The adverse effects of pharmaceuticals in the environments are recognized by numerous examples worldwide, by which acute and chronic exposition with non-observed effects of chemicals in waters could not be a negligible fate. Integrated pharmaceutical pollution management can counteract this emerging global problem and minimize wildlife and food contamination exposures, thus decreasing human health risk.

In Brazil, the laws dealing with medical waste management are recent, with gaps that might favor non-compliance with the law by the general public or manufacturers in the pharmaceutical facilities and industry. Pharmacies and drugstores are uncompelled to receive unneeded household medicines, except for those located in cities with over a hundred thousand inhabitants. Thus, in general, in small cities, the management and disposal of drug residues are still in a personal, non-oriented and practically dysregulated form, disconnected of strict laws and collective directives. In contrast to the Federation, Brazilian State laws that establish pharmacies and drugstores should, regardless of whether they are public or private, compel the collection of expired, unused, or unwanted medicines from patients’ homes and handle these pharmaceutical residues properly. However, the latent problem is the applicability of the legislation and the dissemination of good practice among the co-participants. In fact, up to the present, the collection and handling by privates or public drugstores are incipient and do not comply with the actual necessity of controlling pharmaceuticals’ release in the environment. Considering the expanding local pharmaceutical industry and trade, PCPPs’ consumption and APIs discharge in wastewaters and soil by a growing population with restrictive WWTP output might compromise the present and future environment. Thus far, the entire community’s engagement is and will be required, as well as nationwide uniform directives, for a safe, eco-friendly environment and sustainable society.

## 5. Future Directions

Based on the current knowledge and the Brazilian context, in Table 4, we summarize some concerns about APIs and PCPPs entering the environment, annotate the flaws in the local legislation to overcome, and indicate several scientific and technological measures that could control pharmaceutical/drug pollution.

## Figures and Tables

**Table 1:** Medicines and pharmaceutical products with the highest sales in Brazil, according to their active ingredient(s) and therapeutic indication(s).

Ranking	Pharmaceuticals Products	Therapeutic Indication
1	Dorflex^®^ (dipyrone monohydrate, orphenadrine citrate, and caffeine)	To relieve pain associated with muscle contractures, including tension headache
2	Xarelto^®^ (rivaroxaban)	To prevent venous thromboembolism in adult patients undergoing elective knee or hip arthroplasty surgery
3	Saxenda^®^ (liraglutide)	Chronic weight control in adults with a Body Mass Index of 27 kg/m^2^ or more
4	Neosaldina^®^ (dipyrone, isometheptene mucate, and anhydrous caffeine)	To treat various types of headache, including migraines, or for the treatment of colic
5	Addera D3^®^ (cholecalciferol)	Auxiliary treatment of bone demineralization (removal of minerals) before and after menopause, rickets, osteomalacia, osteoporosis and in the prevention of falls and fractures in older adults with vitamin D deficiency
6	Glifage XR^®^ (metformin hydrochloride)	To treat type 2 diabetes in adults, alone or in combination with other oral anti-diabetics. Also used to treat type 1 diabetes in addition to insulin therapy and indicated to treat Polycystic Ovary Syndrome
7	Torsilax^®^ (caffeine, carisoprodol, diclofenac sodium and paracetamol)	To treat rheumatism in its acute and chronic inflammatory-degenerative forms: acute gout crisis, acute inflammatory, post-traumatic and post-surgical states, acute exacerbations of rheumatoid arthritis or other rheumatic arthropathies, osteoarthritis, and acute states of rheumatism in extra-articular tissues, low back pain or low back pain. Also, an adjunct to severe inflammatory processes resulting from infectious conditions
8	Victoza^®^ (liraglutide)	Chronic weight control in adults with a Body Mass Index (BMI) of 27 kg m^-2^ or more
9	Anthelios^®^ (avobenzone, homosalate, octisalate, octocrylene and oxybenzone)	Sun protection
10	Puran T-4^®^ (sodium levothyroxine)	In patients with hypothyroidism of any etiology (except for transient hypothyroidism, during the recovery phase of subacute thyroiditis), a replacement therapy or hormonal supplementation. Suppression of pituitary TSH in the treatment or prevention of various euthyroid goiter types, including thyroid nodules, subacute or chronic lymphocytic thyroiditis (Hashimoto’s thyroiditis), thyroid-dependent follicular and papillary carcinomas. Upon diagnosis in suppression tests, aiding in the diagnosis of suspected mild hyperthyroidism or an autonomous thyroid gland.
11	Selozok^®^ (metoprolol succinate)	Arterial hypertension: reduction in blood pressure, morbidity, and risk of mortality from the cardiovascular and coronary origin (including sudden death); Angina pectoris; Adjuvant in the therapy of symptomatic chronic heart failure, mild to severe; Changes in heart rhythm, including especially supraventricular tachycardia; Maintenance treatment after myocardial infarction; Functional cardiac changes with palpitations; Migraine prophylaxis
12	Aradois^®^ (losartan potassium)	To treat hypertension and the treatment of heart failure when therapy with ACE inhibitors is no longer adequate.
13	Sal de Eno^®^ (sodium bicarbonate, sodium carbonate and citric acid)	Relief from heartburn, poor digestion, and other stomach disorders, such as excess stomach acid and acid indigestion
14	Novalgina^®^ (dipyrone monohydrate)	Analgesic and antithermic
15	Jardiance^®^ (empagliflozin)	Diabetes mellitus type 2
16	Alenia^®^ (budesonide and formoterol)	To improve and control shortness of breath in patients with bronchoconstriction or bronchospasm in patients with bronchial asthma.
17	Prolopa^®^ (levodopa and benserazide)	Parkinson’s disease
18	Galvus Met^®^ (vildagliptin and metformin)	Diabetes mellitus type 2
19	Ninho Fases 1+^®^ (milk infant formula)	Food supplement
20	Venvanse^®^ (tablisdexanfetamine)	Attention deficit hyperactivity disorder.

Source: Interfarma, 2019; Sanofi, 2014; Bayer, 2018; Novo Nordisk, 2016; Takeda Pharma Ltd., 2013; Mantecorp Farmasa, 2019; Merck S/A, 2014; Neo Química, 2019; Novo Nordisk, 2014; Sanofi, 2020; AstraZeneca, 2018; Biolab, 2019; GlaxoSmithKline Brasil, 2019; Sanofi, 2017; Boehringer Ingelheim, 2018; Biosintetica, 2014; Produtos Roche Químicos e Farmacêuticos S.A., 2019; Novartis, 2018; Shire, 2019.

**Table 2:** Some reported APIs and PCPPs found in water bodies and sediment in Brazilian territory.

Environment Compartment	Sampling Locality (Brazilian State)	API/PCPP Pollutants (Mean or Range of Concentration)	Refs.
Water Reservoir	Water source (SP)	acetominophen (0.03 μg L^−1^), benzophenone-3 (170.87 μg L^−1^), diclofenac (0.02 μg L^−1^), ibuprofen (0.01 μg L^−1^), methylparaben (1.14 μg L^−1^), naproxen (0.01 μg L^−1^),	[42]
River (Surface Water)	Urban water (SP)	norfloxacin (8–18 ng L^−^^1^)	[43]
River (Surface Water)	Lagoon Complex (RJ)	acetaminophen (0.09–0.14 μg L^−1^), bisphenol a (0.22 μg L^−1^), diclofenac (1.37–39.86 μg L^−1^), salycilic acid (1.65–4.81 μg L^−1^)	[44]
River (Surface water)	Urban stream (SP)	atenolol (1182 ng L^−^^1^), caffeine (14955 ng L^-1^), carbamazepine (71.9 ng L^−^^1^), diclofenac (92.6 ng L^−^^1^), 17-α-ethinylestradiol (<0.16 ng L^−^^1^), 17-β-estradiol (1.85 ng L^−^^1^), estrone (6.90 ng L^−^^1^), ibuprofen (185.3 ng L^−^^1^), naproxen (103.7 ng L^−^^1^), paracetamol (3702 ng L^−^^1^), propranolol (15.2 ng L^−^^1^), triclosan (35.2 ng L^−^^1^)	[45]
River (Surface Water)	Suburban water (MG)	bisphenol A (8.6–168.3 ng L^−^^1^), diethyl phthalate (5.0–410.9 ng L^−^^1^), 17-α-ethynylestradiol (5.6–63.8 ng L^−^^1^), 17-β-estradiol (5.6–63.8 ng L^−^^1^), nonylphenol (25.9–1435.3 ng L^−^^1^)	[46]
Tap water	Source/drinking water (SP)	Cocaine (6–62 ng L^−^^1^), benzoylecgonine (10–1019 ng L^−^^1^)	[47]
Coastal water	Urban surface runoff (SP)	acetaminophen (18.3–391.0 ng L^−^^1^), atenolol (0.1–140.0 ng L^−^^1^), benzoylecgonine (0.9–278.0 ng L^−^^1^), carbamazepine (0.1–8.0 ng L^−^^1^), chlortalidone (0.1–0.4 ng L^−^^1^), citalopram (0.2–0.4 ng L^−^^1^), clopidogrel (0.1–0.2 ng L^−^^1^), cocaine (0.2–30.3 ng L^−^^1^), diclofenac(0.9–79.8 ng L^−^^1^), enalapril (2.2–3.8 ng L^−^^1^), losartan (3.6–548.0 ng L^−^^1^), orphenadrine (0.2–1.5 ng L^−^^1^), rosuvastatin (2.5–38.5 ng L^−^^1^), valsartan (19.8–798.0 ng L^−^^1^)	[48]
Amazon wetland	Surface water and sediment (MA)	Surface water: acetaminophen (455–1716 ng L^−^^1^), albendazole (<4–22 ng L^−^^1^), caffeine (29–7940 ng L^−^^1^), carbamazepine (7–3 ng L^−^^1^), diclofenac (<100–463 ng L^−^^1^), ethylparaben (<52 ng L^−^^1^), furosemide (<52–112 ng L^−^^1^), ibuprofen (<100–320 ng L^−^^1^), lidocaine (<20–41 ng L^−^^1^), mebendazole (4–18 ng L^−^^1^), methylparaben (<20–660 ng L^−^^1^), sulfamethoxazole (<20–120 ng L^−^^1^)Sediment: albendazole (1–13 ng g^−1^), avobenzone (51 ng g^−1^), benzophenone-3 (<3–17 ng g^−1^), caffeine (6–20 ng g^−1^), enalapril maleate (1 ng g^−1^), ketoconazole (<5–277 ng g^−1^), mebendazole (<1–4 ng g^−1^), methylparaben (<5–14 ng g^−1^), nifedipine (75–105 ng g^−1^), propranolol (2–2 ng g^−1^), triclocarban (<1–1318 ng g^−1^), triclosan (50–137 ng g^−1^)	[49]
Marine sediment	Submarine sewage outfalls (SP)	nonylphenol (13.3 to 72.5 ng g^−1^), octylphenol (49.2 ng g^−1^), triclosan (3.3 ng g^−1^)	[50]
Marine sediment	Watershed, Bay (BA)	atenolol (0.48–9.84 ng g^−1^), carbamazepine (<0.10–4.81 ng g^−1^), diazepam (<0.10–0.71 ng g^−1^), diclofenac (<0.10 to 1.06 ng g^−1^), erythromycin (<0.10–2.29 ng g−1), ibuprofen (0.77–18.8 ng g^−1^),	[51]
Wastewater Effluent	Urban catchments (RS)	ibuprofen (0.5 μg L^−1^–1.26 μg L^−1^), paracetamol (0.4 μg L^−1^–3.0 μg L^−1^)	[52]
Hospital Effluent	University Hospital (RS)	bromazepam (137–195 ng L^−1^), carbamazepine (461 ng L^−1^–590 ng L^−1^), clonazepam (57 ng L^−1^–134 ng L^−1^), diazepam (571 ng L^−1^–641 ng L^−1^), lorazepam (42 ng L^−1^–96 ng L^−1^),	[35]
WWTPs (Influent)	Metropolitan area (MG)	estriol (17.1 μg L^−1^–148.8 μg L^−1^), estrone (3.3 μg L^−1^–5.4 μg L^−1^), triclosan (0.72 μg L^−1^–7,42 μg L^−1^),	[53]
WWTPs (influent)	Raw sewage (MG)	bezafibrate (94.4 ng L^−1^), diclofenac (99.9 ng L^−1^), sulfamethoxazole (13.0 ng L^−1^), trimethoprim (61.5 ng L^−1^)	[54]

Pharmacological category/application of APIs and PCPPs: analgesics (acetaminophen/paracetamol, ibuprofen), antibiotics (erythromycin, trimethoprim, sulfamethoxazole, salicylic acid), antihypertensive (atenolol, enalapril, losartan, nifedipine, valsartan), anticholesterolemic/lipidemic (losartan, rosuvastatin, bezafibrate), antidepressant (citalopram), antifungal (ketoconazole), anthelmintic (albendazole, mebendazole), anti-inflammatory (diclofenac, naproxen, orphenadrine), antiplatelet (clopidogrel), antiseptic (triclocarban, triclosan), non-ionic surfactants alkylphenol ethoxylates and metabolites (nonylphenol, octylphenol), diuretic (chlortalidone), plasticizer (bisphenol A, diethyl phthalate), natural estrogens (estrone, 17-β-estradiol, estriol), estrogenic contraceptive (17α-ethynylestradiol), illicit drug and metabolite (cocaine, benzoylecgonine), preservatives (benzophenone-3, ethylparaben, methylparaben), psychoactive drugs (bromazepam, carbamazepine, clonazepam, diazepam, lorazepam), stimulant (caffeine), sunscreen (avobenzone).

**Table 3:** Active pharmaceutical ingredients contained in medicines recommended being flushed into the toilet according to U. S. FDA.

Active Pharmaceutical Ingredient
Fentanyl and Fentanyl citrate
Morphine Sulfate
Buprenorphine Hydrochloride (+Naloxone Hydrochloride)
Buprenorphine
Methylphenidate and Meperidine Hydrochloride
Diazepam
HydromorphoneHydrochloride
Methadone Hydrochloride
Morphine sulfate
Tapentadol
Oxymorphone Hydrochloride
Oxycodone Hydrochloride
Acetaminophen (+Oxycodone Hydrochloride)
Aspirin (+Oxycodone Hydrochloride)
Sodium Oxybate
Hydrocodone Bitartrate

Adapted from the U.S. FDA “Flush List” [74].

**Table 4:** Some concerns and recommendations about pharmaceutical/drug pollution in the Brazilian context.

Main concerns - Flaws in the legislation and regulations that preclude strict compliance of consumers and manufacturers - Lack of educational and official programs to collect expired, unused, and unwanted medicines, such as take-back program and reverse logistic; - Lack of a local, official flush list and directions for solid-residues disposal for useless medicines - Public unawareness of the increasing pharmaceutical/drug pollution and its ecotoxicological effects - Lack of legislation that regulates the MCL of APIs and PCPPs in drinking water and the environment. - The low number of installed WWTPs and variable efficiency of WWTPs in eliminating APIs and PCPPs from domestic, industrial, and hospital sewages - Use o biosolids to fertilize crops in extensive agriculture that disperse APIs and PCPPs in the soil - Absence of systematic analytical programs to assess the level of pharmaceutical pollution nationwide
Recommendations - Fill the gap of imperfect legislation and regulation about the proper disposal of medicines - Uniformize the directives nationwide on the disposal of medicines for human and veterinary uses - Adopt national take-back programs and reverse logistic strategies to collect useless medicines and avoid pharmaceuticals ending in the environment - Update the directives by taking into account the PBT index of pharmaceuticals and chemicals - Conduct consistent and periodical ecological risk assessment to nurture policies of drug pollution; - Aware the general public about the harmful effects on human health and wildlife regarding the presence of APIs and PCPPs in the environment - Orientate consumers about the proper way to discard or return useless medicines - Educate students of all academic levels about the short- and long-term detrimental effects of APIs and PCPPs on the Earth biome - Establish regular monitoring program using high sensitive analytical procedures to detect APIs, PCPPs, and metabolites in critical water systems - Adopt biological/ecological filtration to treat wastewater before discharge into the water stream - Adopt advanced oxidative processes (e.g., UV/H_2_O_2_) and membrane separation (e.g., reverse osmosis) for APIs and PCPPs removal in WWTPs

Note: MCL, maximum contaminant level; UV, ultraviolet;

## Data Availability

Data sharing not applicable.

## Supplementary Materials

The following are available online at https://www.mdpi.com/article/10.3390/jox11020005/s1, Supplementary Table S1.
