# Supplementary material for: Pharmaceutical Pollution and Disposal of Expired, Unused, and Unwanted Medicines in the Brazilian Context
Source: J Xenobiot. 2021 May 18;11(2):61–76. doi: 10.3390/jox11020005 (PMC8162542; doi:10.3390/jox11020005)
Supplement: Supplementary file 1 [file jox-11-00005-s001.zip › jox-1201845-supplementary.pdf]

Supplementary Table 1. Leading issues in Brazilian State laws on overdue drug disposal by regions.

Regions of Brazil	State Legislation	Description
**North**		
Acre	Law nº 2720 of 07/25/2013	It creates the State Program for the Collection of Expired or Spoiled Medicines.
Amapá	Bill nº 0116/12-AL of 05/23/2012	It creates the State Program for the Collection of Expired or Spoiled Medicines and provides other measures.
Amazonas	Law nº 3.676 of 12/12/2011	It creates the State Program for the Collection of Expired or Spoiled Medicines.
	Law nº155 of 07/12/2013	It provides for the pharmaceutical industries and drug distribution companies' responsibility to properly dispose of drugs with expired expiration dates and adopt other measures.
Pará	---	---
Rondônia	---	---
Roraima	---	---
Tocantins	Bill nº232 of 12/21/2017 and Bill nº68 of 04/20/2017	It obliges pharmacies and drugstores in the State of Tocantins to keep containers for collecting medicines, cosmetics, pharmaceutical supplies, and related items that have deteriorated or have expired and gives other measures.
**Northeast**		
Alagoas	Ordinance nº63 of 02/19/2015	It officializes the disposal and donation of medicines and related products with an expiration date close to expiration.
Bahia	Law nº14123 of 09/12/2019	It obliges pharmacies and drugstores within the State of Bahia's scope to keep containers for collecting expired medicines, cosmetics, pharmaceutical supplies, and related items and takes other measures.
Ceará	Law nº15934 of 12/29/2015	It amends article 1 of law nº 15192, of July 19 of 2012, which defines rules for the disposal of expired and/or discontinued drugs.
	Law nº 15192 of 07/19/2012	It defines rules for the disposal of expired and/or discontinued drugs.
Maranhão	Law nº 11247 of 03/27/2020	It institutes the State Awareness Campaign for the Correct Disposal of Expired and/or Discontinued Medicines and takes other measures.
	Law nº 9727 of 12/11/2012	It obliges manufacturers and drug distribution companies to proceed to the selective collection and proper destination of expired drugs, implement an information policy on the risks caused by these products within the State, and take other measures.
Paraíba	Law nº 9646 of 12/29/2011	It provides for the rules for the final destination of the disposal of expired or unfit medicines for use within the scope of the State of Paraíba, and it provides other measures.
Piauí	---	---
Pernambuco	Manual for disposal of solid waste from the State Secretariat for the environment (2017)	Guide consumers on how and where to dispose of solid waste in Pernambuco.
	Bill nº 596 of 11/16/2015	It creates the State Medicines Disposal Program, its environmentally appropriate final destination, and takes other measures.
	Bill nº163 of 04/12/2019	Within the scope of the State of Pernambuco, it institutes the reverse

		logistics of medicines discarded by the consumer and takes other measures.
Rio Grande do Norte	Law nº10094 of 08/04/2016	It provides for collecting and disposing of expired medicines in the State of Rio Grande do Norte and provides other measures.
Sergipe	Law nº7913 of 11/03/2014	Pharmacies and drugstores in the State of Sergipe are obliged to keep containers for collecting medicines, cosmetics, pharmaceutical supplies, and related items damaged or expired.
**Midwest**		
Distrito Federal	Law nº5092 of 05/04/2013	It provides for the obligation of pharmacies and drugstores to receive expired medicines for disposal.
Goiás	Law nº19462 of 10/11/2016	It provides for the collection and disposal of expired medicines and other measures.
Mato Grosso	Law nº10600 of 09/26/2017	It obliges pharmacies to receive expired drugs and pharmaceuticals and takes other measures.
Mato Grosso do Sul	Law nº5180 of 04/12/2018	It amends Law No. 4474 of 03/06/2014.
	Law nº4474 of 03/06/2014	It provides pharmacies and drugstores' obligation to keep containers for collecting medicines, cosmetics, and pharmaceutical and related supplies deteriorated or with an expired expiration date.
**Southeast**		
Espírito Santo	Law nº8454 of 04/16/2013	It institutes the "Responsible Disposal" Campaign.
	Law nº10994 of 05/27/2019*	It obliges everyone involved in the pharmaceutical production chain in general to the structure. It implements reverse logistics systems so that these substances and their packaging are directed to the collection in previously established locations, independently of the public service of urban cleaning and solids waste management.
Minas Gerais	---	---
Rio de Janeiro	Bill nº 4147 of 05/29/2018 and Bill nº 1263 of 02/07/2012	It provides for the collection and disposal of expired or used medicines in the State of Rio de Janeiro and provides other measures.
	Law nº 8135 of 10/15/2018	It institutes the state awareness campaign for the correct disposal of expired and/or discontinued drugs.
São Paulo	Law nº12300 of 03/16/2006	It institutes the State Solid Waste Policy and defines principles and guidelines
**South**		
Paraná	Law nº17211 of 03/07/2012	This law provides for disposing of medicines in disuse in the State of Paraná and its procedures.
	Decree nº9213 of 10/23/2013	It regulates Law No. 17211, of July 3, 2012.
Rio Grande do Sul	Law nº13905 of 01/10/2012	It provides pharmacies and drugstores' obligation to keep containers for collecting medicines, cosmetics, pharmaceutical supplies, and related items deteriorated or with an expired date.
	Law nº15339 of 10/02/2019	It institutes the Solidary Program - Pharmacy Solidarity - awareness, donation, reuse, dispensation for the population and disposal of medicines within the State of Rio Grande do Sul and other measures.
Santa Catarina	Bill nº0056 of 03/10/2016	It institutes the State Policy for Reverse Logistics of Medicines within the State of Santa Catarina's scope.

	Law nº13557 of 11/17/2005	It provides with the State Solid Waste Policy and adopts other measures.

* Rule with effectively suspended by the Espírito Santo State of Court of Justice, under the justification of Direct Unconstitutionality.

--- No state legislation about the disposal of expired or unused drugs. Sources: Acre, 2013; Amapá, 2012; Amazonas, 2011; Amazonas, 2013; Tocantins, 2017; Alagoas, 2015; Bahia, 2019; Ceará, 2015; Ceará, 2012; Maranhão, 2020; Maranhão, 2012; Paraíba, 2011; Pernambuco, 2015; Secretaria de Meio Ambiente e Sustentabilidade, 2017; Pernambuco, 2019; Pernambuco, 2017; Rio Grande do Norte, 2016; Sergipe, 2014; Distrito Federal, 2013; Goiás, 2016; Mato Grosso, 2018; Mato Grosso, 2014; Espírito Santo, 2013; Espírito Santo, 2019; Rio de Janeiro, 2018; Rio de Janeiro, 2012; São Paulo, 2006; Paraná, 2012; Paraná, 2013; Rio Grande do Sul, 2019; Santa Catarina, 2016; Santa Catarina, 2005.
